# Prenatal exposure to metal mixture and sex-specific birth outcomes in the New Hampshire Birth Cohort Study

**DOI:** 10.1097/EE9.0000000000000068

**Published:** 2019-09-12

**Authors:** Antonio J. Signes-Pastor, Brett T. Doherty, Megan E. Romano, Kelsey M. Gleason, Jiang Gui, Emily Baker, Margaret R. Karagas

**Affiliations:** Department of Epidemiology, Geisel School of Medicine, Dartmouth College, Lebanon, New Hampshire.

**Keywords:** in utero exposure, metal mixture, arsenic, manganese, lead, newborns’ size, sex

## Abstract

**Background:**

In utero exposure to individual metals may impact fetal growth, though little is known about the effects of exposure to metal mixtures. Therefore, we investigated joint effects of in utero exposure to a mixture of As (arsenic), Mn (manganese), and Pb (lead) on newborn outcomes in a United States population.

**Methods:**

Concentrations of As, Mn, and Pb were determined in maternal postpartum toenail samples, a biomarker of in utero exposure, from 989 maternal-infant pairs (492 females and 497 males). Newborns’ anthropometric characteristics, including head circumference, length, and weight, were obtained from medical records. The joint effects of the three metals were modeled using Bayesian kernel machine regression and linear regression. Both sex-combined and sex-stratified statistical analyses were performed.

**Results:**

We observed an inverted-U-shape association between maternal toenail Mn concentrations and newborn head circumference, particularly among female infants. Concentrations of Pb were related to reduced head circumference, weight, and length, especially among females at lower concentrations of the other metals. Overall, toenail As concentrations were related to reduced head circumference, especially among males, and an increase in birth length and weight among females. We found little evidence of As interactions with other metals within the mixture.

**Conclusions:**

Our findings suggest that in utero metal mixture exposures may influence birth outcomes, and that such relations may differ by infant sex.

## Abstract 2

Supplemental Digital Content is available in the text.

**What this study adds**

Humans are exposed simultaneously to a variety of toxic metals and metalloids from the environment such as As, Mn, and Pb, which are capable of crossing the placenta, raising concerns regarding fetal growth and development that may impact infants’ lifelong health. Despite the many studies of As, Mn, and Pb as single exposure, we currently lack information regarding the effects of metal mixture exposure during pregnancy. Our findings provide new evidence of the joint effects of in utero exposure to a mixture of As, Mn, and Pb at relatively low levels found in a United States population on newborns’ size.

## Introduction

Humans are exposed simultaneously to a variety of toxic metals and metalloids (referred to as “metals”) through food, water, and airborne sources, as yet little is known about the health effects of exposure to metal mixtures, and whether they differ when assessed as single exposure.^1–3^ Gestation is a particularly vulnerable period of development, and exposures to environmental pollutants during this period may impact infants’ lifelong health.^4–8^ Arsenic (As), manganese (Mn), and lead (Pb) are naturally occurring metals prevalent in the environment including food and drinking water systems, and their ability to cross the placenta has raised health concerns about their impact on the developing fetus that may lead to health effects later on.^9–12^ In addition to its normal physiologic function, the placenta can help to protect the fetal from environmental contaminants and that ability may differ by sex.^13^ Further, sex differences in patterns of exposure, gastrointestinal absorption, metabolism, and detoxification also supports the potential for sex-specific susceptibility to metals toxicity.^14–16^ Studies on sex-specific effects of exposure to metals have not received sufficient attention.^13,14^

Available evidence suggests that in utero exposure to As, Mn, and Pb can affect fetal growth, an important predictor of neonatal mortality and determinant of infant and childhood morbidity.^7,17–25^ Inorganic arsenic, including arsenite and arsenate, is classified as a group I human carcinogen, and exposure to inorganic arsenic has also been associated with a wide array of noncancer endpoints.^26^ Mn is an essential micronutrient and sufficient intake of Mn is typically provided through the diet, making Mn deficiency rare; however, Mn overexposure carries potential toxicity for humans, and the central nervous system is considered the most susceptible target.^27–29^ Pb is considered potentially carcinogenic and is a known neurotoxin.^30–34^ Pb also has been associated with a wide range of other adverse outcomes, including reduced fetal growth.^35,36^ Despite the many studies of As, Mn, and Pb as single exposure, we currently lack information regarding their effects as a metal mixture during pregnancy, especially among populations exposed to relatively low levels. Such effects can now be estimated by applying recently developed statistical approaches designed to accommodate multi-pollutant correlated exposures with the ability to assess nonlinear effects and interactions among mixture components.^37,38^ Thus, we investigated the joint effect of in utero exposure to a mixture of As, Mn, and Pb on newborns’ anthropometric characteristics including head circumference, length and weight, and examined these associations overall and according to infant sex in a United States population drinking from private water systems with unregulated contaminant concentrations.

## Methods

### Study population

Participants were part of the New Hampshire Birth Cohort Study (NHBCS), an ongoing prospective study of over 2,000 participants to date, that was originally designed to examine how pollutants such as metals exposure particularly through drinking water and food affect the health of pregnant women and their children. Beginning in 2009, the NHBCS recruited pregnant women of 18–45 years of age between 24 and 28 weeks of gestation from prenatal clinics in the state of New Hampshire. Eligibility criteria included English literacy, the use of a private, unregulated water system at home (e.g., private well), not planning to move during pregnancy, and singleton births among others described previously.^7,39^ The United States Environmental Protection Agency identifies contaminants in drinking water to protect public health; however, the Environmental Protection Agency does not regulate private wells, and thus consuming water from private wells is an important potential source of exposure to environmental pollutants particularly metals in the state of New Hampshire.^40–42^ Participants provided written informed consent in accordance with the guidelines from the Committee for the Protection of Human Subjects at Dartmouth College.

### Arsenic, manganese, and lead exposure

Toenails are considered a long-term environmental exposure biomarker owing to their slow growth rate and ability to accumulate metals.^41,43,44^ We used As, Mn, and Pb concentrations in maternal toenail samples collected at the mean (SD) of 5.9 (7.1) weeks postpartum as a biomarker of in utero exposure. This measurement reflects exposures occurring several months earlier since toenails grow an estimated 0.03–0.05 mm per day.^43^ Paired toenail samples collected during pregnancy at ~24–28 gestational weeks were also collected to reflect exposure during early pregnancy. Women were provided with detailed instructions to collect toenail clipping samples in paper envelopes after bathing and removing any visible dirt. Five additional washes were performed in an ultrasonic bath using Triton X-100 (LabChem, Pittsburgh, Pennsylvania) and acetone followed by deionized water at the laboratory. Then, we weighed the samples and digested them in Optima nitric acid (Fisher Scientific, St. Louis, Missouri) via low-pressure microwave digestion at the Dartmouth College Trace Element Analysis Core Laboratory. After digestion, final sample weight was recorded and samples were analyzed for As, Mn, and Pb concentrations using inductively coupled plasma mass spectrometry (ICP-MS) on an Agilent 7700× (Agilent Technologies Headquarters, Santa Clara, California).^4^ In each analysis batch, we included duplicate analysis of digested toenail samples and spikes of digested samples, along with blank and fortified blank digests, as quality control measures. Although there is no available toenail As, Mn, and Pb Certified Reference Material yet, the Core participates in a proficiency-testing program (QEMQAS, Center for Toxicology, Quebec, Canada) where hair, which is formed of the same keratinous tissue as nails,^45^ is one of the sample types. The Core laboratory results for As, Mn, and Pb in hair range from 90% to 100% relative to the consensus mean from all the participants of the proficiency-testing program. The median limits of detection (LOD) for toenail As, Mn, and Pb concentrations across batches were 0.020, 0.050, and 0.001 μg/g, respectively. The LOD/sqrt(2) value was assigned for statistical analyses and plots when concentrations were below the LOD.^46^

### Birth outcomes

We obtained infant’s head circumference (cm), length (cm), and weight (g) at birth from their newborn medical records.

### Covariates

A priori, we selected the following covariates often used to assess the association between exposure to environmental pollutants and birth-related outcomes as potential confounders in our models: maternal age at enrollment (years, continuous), smoked cigarette during pregnancy (yes vs. no), maternal highest attained level of education (less than 11^th^ grade or high school graduate or equivalent, junior college graduate or some college or technical school, college graduate, and any post-graduate schooling), maternal body mass index (BMI in kg/m^2^, continuous), and infants’ sex (females vs. males).^47–50^ Adjusting for potential intermediate factors that lie on the pathway from an exposure to a perinatal outcome such as gestational age may lead to biased and paradoxical results,^51^ therefore, we did not include gestational age as a confounding factor in our models. Mothers completed prepartum and postpartum questionnaires about demographics and lifestyle characteristics. We calculated maternal BMI using maternal pre-pregnancy weight combined with height, obtained from medical records. We also identified infant sex from medical records.

### Statistical analysis

For all statistical analyses, we excluded observations with missing values for the covariates. The correlation between concentrations of each metal pair in postnatal toenail samples was examined using Spearman’s correlation coefficients. The Spearman’s correlation coefficients (ρ) were also calculated between pair prenatal and postnatal toenail samples metal concentrations to assess temporal stability of the exposure throughout the pregnancy period. The histograms of toenails As, Mn, and Pb concentrations displayed highly right-skewed distributions, and thus we applied a natural-log transformation (ln) for further statistical analysis. Data on newborns’ head circumference, length, and weight each followed an approximately normal distribution.

We performed Bayesian kernel machine regression (BKMR) primarily as an exploratory method to investigate interactions and joint effects of our metal mixture.^52^ Our BKMR models were specified as Y_i_ = h(As_i_, Mn_i_, Pb_i_) + β^T^ Z_i_ + e_i_, where Y is the continuous infants’ birth outcomes of interest (head circumference, length, or weight); h() is an exposure-response function that accommodates nonlinearity and interactions among mixture components; As, Mn, and Pb are the centered ln concentrations of As, Mn, and Pb in maternal postnatal toenails, respectively; Z are the selected covariates and β are the corresponding regression coefficients. We used the R package “bkmr” to perform the BKMR analysis.^52^ The models included 10,000 Markov chain Monte Carlo iterations using the Gaussian kernel. Then, we performed covariate-adjusted linear regression analyses to further evaluate and quantify the effects of in utero exposure to metals and infant birth outcomes. The linear models included centered ln maternal postnatal toenail As, Mn, and Pb concentrations normalized by their interquartile range (IQR) as independent variables, and newborns’ anthropometric characteristics as the dependent variables (head circumference and length in cm, and weight in g). We first fitted models without interactions between the independent variables adjusting for covariates. Then, we included two-way and three-way interactions of metal ln concentrations, also centered and normalized by their IQR. We performed both sex-combined (adjusted for sex) and sex-stratified analyses of all models.

## Results

### Study population characteristics

Of 1,013 participants whose toenail samples were analyzed for As, Mn, and Pb concentrations and for whom details of their child’s size at birth were available, we ultimately included 989 (97.6%) mother-child pairs with complete covariate data. The covariates with missing values were maternal highest attained level of education (*n* = 21), maternal BMI (*n* = 7), and smoking during pregnancy (*n* = 7). Some of them had missing values in multiple covariates; overall, 24 participants were excluded. Our dataset was evenly distributed among female (*n* = 492) and male (*n* = 497) infants.

Maternal postnatal toenail As, Mn, and Pb concentrations were positively correlated (ρ from 0.27 to 0.46) (Figure S1; http://links.lww.com/EE/A59), with median (IQR) concentrations of 0.05 (0.04–0.09), 0.32 (0.18–0.62), and 0.11 (0.06–0.22) µg/g, respectively (Table 1). In paired toenail samples collected during pregnancy and postpartum a positive correlation was found between each metal concentration (ρ from 0.53 to 0.65) (Figure S2; http://links.lww.com/EE/A59). Mothers were enrolled at the median age of 31 years, and over 70% of them were college graduate or had any post-graduate schooling. The selected characteristics of the study population were similar between male and female infants, including postnatal toenail metal concentrations. However, males had higher head circumference, length, and weight than female infants (Table 1) with a *P*-value < 0.001.

**Table 1:**
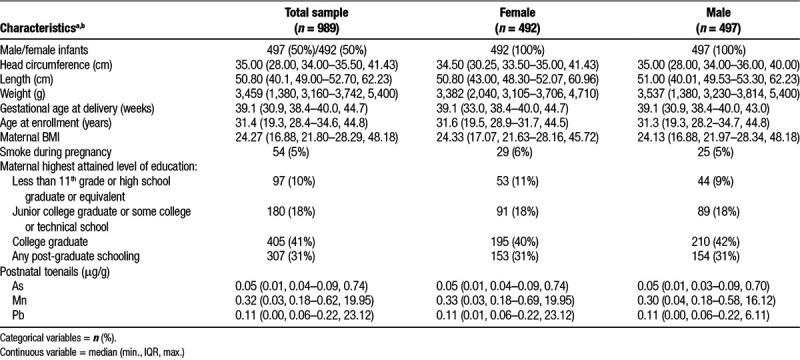
Selected characteristics of the study population for the total sample and stratified by infant sex.

### Bayesian kernel machine regression

**Figure 1.**
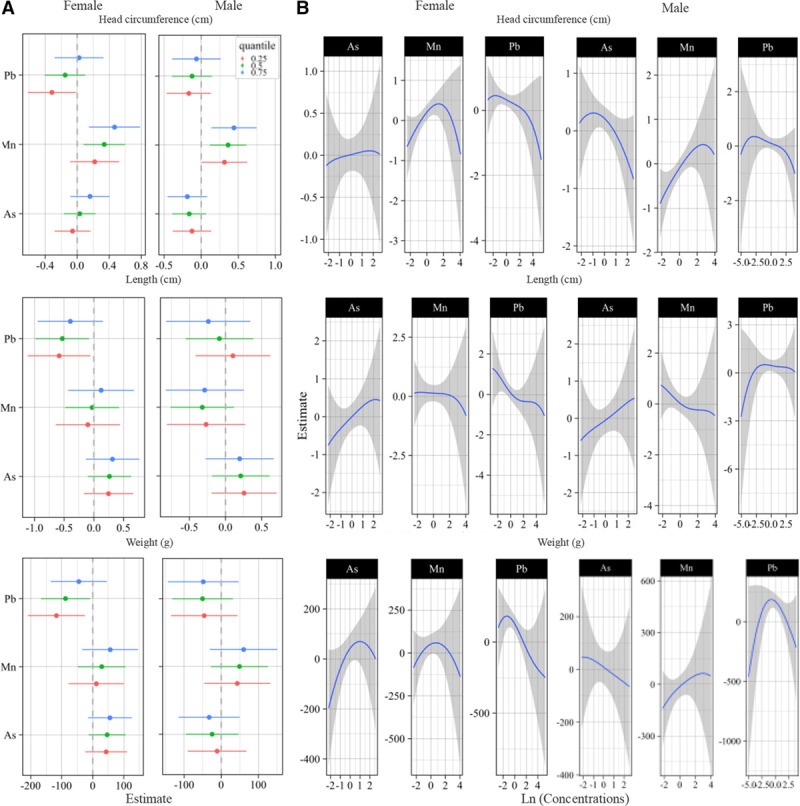
BKMR dose-response functions and interactions within the metal mixture stratified by sex. Models adjusted for maternal age of enrollment (years, continuous), smoked cigarette during pregnancy (yes vs. no), maternal highest attained level of education (less than 11^th^ grade or high school graduate or equivalent, junior college graduate or some college or technical school, college graduate, and any post-graduate schooling), and maternal BMI (kg/m^2^, continuous). A, Single pollutant association (estimates and 95% credible intervals, gray dashed line at the null). This plot compares infants’ size at birth when a single pollutant is at 75^th^ versus 25^th^ percentile, when all the other exposures are fixed at either the 25^th^, 50^th^, or 75^th^ percentile. B, Univariate exposure-response functions and 95% confidence bands for each metal with the other pollutants fixed at the median.

In the BKMR analyses Mn was positively associated with newborns’ head circumference at lower concentrations and negatively associated at higher concentrations (Figure S3B; http://links.lww.com/EE/A59), especially among female infants (Figure 1B); and the positive association appeared stronger at higher percentiles of As and Pb for both sexes (Figure 1A and Figure S3A; http://links.lww.com/EE/A59). In the sex-combined analysis, there was some evidence of nonlinear effects of Pb on newborns’ head circumference, length, and weight (Figure S3B; http://links.lww.com/EE/A59); however, in the sex-stratified analysis, the decrease in head circumference, length, and weight associated to Pb exposure all appeared to be linear, especially at lower percentiles of As and Mn among female infants (Figure 1A and B). Higher toenail As concentrations were associated with a decreased head circumference among males, and an increased length and weight largely among female infants. These associations appeared to follow a linear dose-response function (Figure 1B and eFigure 3B; http://links.lww.com/EE/A59). The effect estimate for an IQR increase in toenail As concentration was consistent across the 25^th^, 50^th^, and 75^th^ percentiles of Mn and Pb concentrations (Figure 1A), suggesting that the effects of As were not modified by the other metals of the mixture.

### Linear regression

Using multiple linear regression analyses, an IQR increase per toenail Mn concentration was associated with a 0.27 cm (95% CI: 0.13, 0.41) increase in newborns’ head circumference (Table 2). In the stratified analysis, an IQR increase per toenail Mn concentration was associated with a 0.22 (95% CI = 0.02, 0.43) and 0.34 (95% CI = 0.15, 0.54) increase in head circumference among females and males, respectively. Also, in the stratified analyses, an IQR increase in toenail As concentration was associated with a −0.20 cm (95% CI = −0.38, −0.02) decrease in head circumference among males (Table 2). Conversely, an IQR increase per toenail As concentration was associated with a 0.31 cm (95% CI = 0.02, 0.60) increase in length, and to a lesser extent with a 47.94 g (95% CI = −5.46, 101.33) increase in weight among female infants. In addition, among female infants, an IQR increase per toenail Pb concentration was associated with a −0.20 cm (95% CI = −0.39, −0.01), and −62.17 g (95% CI = −124.08, −0.25) decrease in head circumference and weight, respectively. We also observed an attenuated decrease in female infants’ length of −0.30 (95% CI = −0.64, 0.03) per IQR increase in toenail Pb concentration. We detected statistically significant three-way metal interactions for length and weight, especially among male infants (Table 2). The main findings from our BKMR and linear regression models stratified by sex are summarized in Table S1 (http://links.lww.com/EE/A59).

**Table 2:**
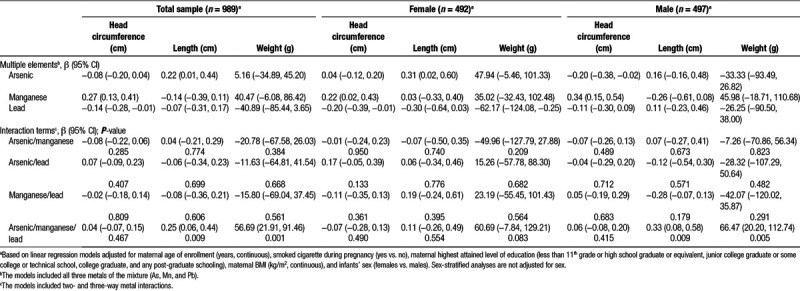
Change in newborns’ head circumference, length, and weight with each IQR increase in maternal toenail metal concentration both sex-combined and sex-stratified.

## Discussion

Our findings, based on a United States population, suggest that in utero exposure to a metal mixture of As, Mn, and Pb may influence fetal growth with differences according to the exposure level of each metal and infant sex. Pb concentrations were related to reduced head circumference, length, and weight among female infants at lower concentrations of the other metals, and the shape of the dose-response curve appeared linear. We observed an inverted-U-shape association between maternal toenail Mn concentrations and newborns’ head circumference, particularly among female infants. Toenail As concentrations were related to reduced head circumference, especially among males, and an increase in birth length and weight largely among female infants, with little evidence of nonlinearity.

We used postpartum maternal toenail metal concentrations as a biomarker of in utero metal exposure. Fibrous proteins (i.e., keratins) in nail tissue are capable of binding metals and are considered a biomarker of long-term exposure to As due to their slow growth.^43,53^ In addition to As, a growing body of evidence suggests that toenail Mn and Pb concentrations may serve as a cumulative exposure biomarker.^41,43–45,53–56^ Toenail metal concentrations reflect exposures occurring several months earlier; however, the exact timing of exposure may vary depending on a variety of factors that may affect toenail growth.^43^ Nevertheless, in our study population, a relatively strong positive correlation was found between each metal concentration in paired toenail samples collected during pregnancy at enrollment and postpartum, suggesting temporal stability of metal exposures throughout the pregnancy period in this cohort.

Prior studies of metal mixtures and birth outcomes have investigated occupationally exposed individuals or those living in contaminated areas.^57–60^ Previous studies also have typically focused on single metal exposures, which do not reflect individuals’ realistic exposure to a mixture of metals.^7,35,61^ Our study examined metal mixture effects at relatively low levels of exposure in association with infants’ size at birth using both a traditional approach and a more recent statistical method to estimate the effects of exposure to a mixture of correlated metals prevalent in the environment.

Applying the BKMR statistical approach enabled us to explore the effects of correlated metal mixtures and allowed for estimations of nonlinear and nonadditive dose-response relationships.^2,52^ Our results from the BKMR analyses suggest an antagonistic effect of Mn and Pb on infant’s head circumference. The BKMR single pollutant association plot indicated an inverse association with Pb particularly at lower levels of the other metals. For Mn a positive trend was observed, especially at higher levels of the other metals. The effect estimates for As did not appear to change with the level of exposure of the other metals of the mixture; however, we found statistically significant three-way metal interactions in the linear regression models, largely among male infants.

Mn is an essential nutrient and as such, is necessary for proper growth and development.^61,62^ Further, it has been suggested that Mn may minimize free radical formation and protect against oxidative damage during pregnancy and thus fetal growth.^62^ A positive association has been found between deciduous tooth Mn and birth weight.^63^ However, evidence suggests that Mn follows a U-shaped dose-response curve, and lower and higher maternal urine and blood Mn levels during pregnancy have been related to lower birth weight.^61,64,65^ Still, the optimal Mn levels have not been defined.^61^ Our findings from the BKMR univariate exposure-response functions suggest that the association between Mn and newborns’ head circumference follows an inverted U-shaped dose-response mainly among female infants; however, there were wide confidence bands at higher levels of exposure due to the few participants at high exposure levels. In contrast, the positive association between Mn and newborns’ head circumference at lower concentrations was more precise and supported by our findings from the linear regression analyses. Prior studies have reported that higher maternal blood Pb concentrations were associated with decreased length and weight at birth in a study in China^35^ and with a decrease in birth weight in the United States.^66^ An inverse association has also been reported between deciduous tooth Pb and newborns’ weight.^63^ Potential mechanisms linking prenatal Pb exposure with impaired fetal growth include competition of Pb with calcium for deposition into bone and impact on collagen synthesis, which may be exacerbated at lower levels of essential nutrients such as Mn.^10,66^

A growing body of evidence suggests sex-related differences in response to in utero exposure to environmental pollutants associated with differences in the structure and function of the placenta.^13,67^ In our analyses, we found evidence that the effects of in utero exposure to As and Pb as part of a metal mixture may be sex-specific. Indeed, our findings suggest that exposure to As during pregnancy was related to an increase in birth length and weight among females, but the later did not reach statistical significance. Conversely, As exposure was associated with a decrease in head circumference among male infants. A prior study from the NHBCS reported a reduction in newborns’ head circumference, and increased length largely in males based on maternal urinary As during pregnancy.^7^ In multivariable regression models adjusted for Pb and Mn, maternal blood As at delivery was associated with a decrease in newborns’ head circumference and weight in a study population residing near a mining-related Superfund site in Northeast Oklahoma.^68^ Further, in a cohort in Bangladesh, maternal As exposure during pregnancy assessed using urinary As concentrations was associated with reduced head circumference based on ultrasounds taken before the third trimester of pregnancy.^21^ Our findings indicate that females may be more susceptible to the effects of Pb compared to male infants. Data regarding sex differences in susceptibility to the effects of metal mixtures on fetal development remains scarce and requires further investigation in large-scale initiatives.

We observed temporal stability of As, Mn, and Pb exposure throughout pregnancy. However, exposure misclassification remains a potential source of bias. Further, we adjusted for several potential confounding factors including maternal age of enrollment, smoking during pregnancy, level of maternal education, maternal BMI, and infant sex, but the effects of unknown factors or residual confounding from other metals remains a possibility. The extent of metal exposure for our study population was lower compared to that reported in previous studies focused on populations occupationally exposed or living in contaminated areas,^57–60^ which limits our ability to detect the presumably more subtle effects of lower dose exposures. Similarly, our sex-stratified findings are based on modest sample sizes and thus could have been due to chance.

In a United States cohort of mothers and infants, our findings suggest that the effect of in utero exposure to metals may influence infant’s birth outcomes and may differ by the exposure level of other metals of the mixture and infant sex.

## Supplementary Material
