# Supplementary material for: Prenatal exposure to metal mixture and sex-specific birth outcomes in the New Hampshire Birth Cohort Study
Source: Environ Epidemiol. 2019 Sep 12;3(5):e068. doi: 10.1097/EE9.0000000000000068 (PMC6914313; doi:10.1097/EE9.0000000000000068)
Supplement: Supplementary file 1 [file ee9-3-e068-s001.docx]

**SUPPORTING CONTENT**

**eFigure 1**: Spearman’ correlation matrix for postnatal toenail metal concentrations in μg/g for the total samples and stratified by sex (male *n* = 497; female *n* = 492; total samples *n* = 989).

**eFigure 2**: Spearman’s correlation between pair pre- and postnatal toenail samples metal concentrations in μg/g (*n* = 922).

**eTable 1**: Summary of the main findings according to sex and applied statistical methodology.

|  | BKMR | Linear regression |
| --- | --- | --- |
| Female | Suggestive evidence of a positive linear effect of **As** on length and to a lesser extent with weight with little evidence of interactions with the other metals of the mixture.  Suggestive evidence of a nonlinear effect of **Mn** on head circumference with a positive more precise association at lower levels that appears stronger at higher concentrations of the other metals of the mixture.  Suggestive evidence of an inverse linear effect of **Pb** on head circumference, length, and weight that appears stronger at lower percentiles of the other metals of the mixture. | Positive association between **As** and length and to a lesser extent with weight.  Positive association between **Mn** and head circumference.  Inverse association between **Pb** and head circumference and weight, and to a lesser extent with length. |
| Male | Suggestive evidence of an inverse linear effect of **As** on head circumference with little evidence of interactions with the other metals of the mixture.  Suggestive trend of a nonlinear effect of **Mn** on head circumference to a lesser extent compared to that for female infants with a positive more precise association at lower levels that appears stronger at higher concentrations of the other metals of the mixture. | Inverse association between **As** and head circumference.   Positive association between **Mn** and head circumference. |

**eFigure 3:** BKMR dose-response functions and interactions within the metal mixture.

Models adjusted for maternal age of enrollment (years, continuous), smoked cigarette during pregnancy (yes *vs.* no), maternal highest attained level of education (less than 11^th^ grade or high school graduate or equivalent, junior college graduate or some college or technical school, college graduate, and any post-graduate schooling), maternal BMI (kg/m^2^, continuous), and infants’ sex (female *vs.* males). (**A**) Single pollutant association (estimates and 95% credible intervals, gray dashed line at the null). This plot compares infants’ size at birth when a single pollutant is at 75^th^ vs. 25^th^ percentile, when all the other exposures are fixed at either the 25^th^, 50^th^, or 75^th^ percentile. (**B**) Univariate exposure-response functions and 95% confidence bands for each metal with the other pollutants fixed at the median.
